# Supplementary material for: Unveiling the chain mediation model of sleep quality and fatigue in linking subjective exercise experience to depression in adolescent
Source: PLoS One. 2026 Apr 1;21(4):e0333460. doi: 10.1371/journal.pone.0333460 (PMC13043057; doi:10.1371/journal.pone.0333460)
Supplement: Attachment — Submitted filename: Response to Reviewer.docx [file pone.0333460.s002.docx]

Dear Editor,

Thank you very much for your thorough consideration for our manuscript (**PONE-D-25-44933**) entitled “**Unveiling the Chain Mediation Model of Sleep Quality and Fatigue in Linking Subjective Exercise Experience to Depression in Adolescent**” for publishing in PLOS One. We have paid our close attention to all comments and suggestions raised by the academic editor and reviewers and responded to all with changes in clarity and explanation. Revisions marked with yellow color have been carefully made to the manuscript and a detailed response is given below.

We hope our replies and revision to the manuscript would satisfy the requirements from you and reviewers. Please feel free to let me know if you have further questions on our revised manuscript. Thank you very much for your consideration and best wishes to you and the reviewers.

We look forward to hearing from you at your earliest convenience.

Sincerely Yours

Wenliang Ji

School of Physical Education

University of Jinan

Jinan 250022, China

E-mail: spe_jiwl@ujn.edu.cn

**Replies to the academic editor**

**1.** **Please ensure that your manuscript meets PLOS ONE's style requirements, including those for file naming. The PLOS ONE style templates can be found at**

**https://journals.plos.org/plosone/s/file?id=wjVg/PLOSOne_formatting_sample_main_body.pdf and**

**https://journals.plos.org/plosone/s/file?id=ba62/PLOSOne_formatting_sample_title_authors_affiliations.pdf**

**Response:** We have carefully revised the manuscript to ensure full compliance with PLOS ONE’s style and formatting requirements, including file naming conventions. The manuscript has been formatted in accordance with the official PLOS ONE templates for the main body and title/authors/affiliations.

**2. PLOS requires an ORCID iD for the corresponding author in Editorial Manager on papers submitted after December 6th, 2016. Please ensure that you have an ORCID iD and that it is validated in Editorial Manager. To do this, go to ‘Update my Information’ (in the upper left-hand corner of the main menu), and click on the Fetch/Validate link next to the ORCID field. This will take you to the ORCID site and allow you to create a new iD or authenticate a pre-existing iD in Editorial Manager.**

**Response:** The corresponding author has now been successfully validated in Editorial Manager via the “Fetch/Validate” function, in accordance with PLOS ONE’s submission requirements.

**3. Thank you for stating the following financial disclosure:“National Social Science Fund of China grant number [21BTY004]” Please state what role the funders took in the study. If the funders had no role, please state: "The funders had no role in study design, data collection and analysis, decision to publish, or preparation of the manuscript." If this statement is not correct you must amend it as needed. Please include this amended Role of Funder statement in your cover letter; we will change the online submission form on your behalf.**

**Response:** Thank you for the request to clarify the role of the funding agency. We have carefully revised the Role of Funder statement to accurately reflect the funder’s contribution. The funding agency provided conceptual guidance and research ideas at the initial stage of the study.

The amended Role of Funder statement is as follows:

The funder contributed to the initial conceptualization of the study by providing research ideas and decisions to publish. The funder had no role in data collection and analysis, interpretation of the results or preparation of the manuscript.

As requested, this statement is included in the present cover letter.

**Please note that funding information should not appear in any section or other areas of your manuscript. We will only publish funding information present in the Funding Statement section of the online submission form. Please remove any funding-related text from the manuscript**

**Response:** All funding-related information has been completely removed from the manuscript text. Funding details are now provided exclusively in the Funding Statement section of the online submission form, in accordance with PLOS ONE policy.

**4. Please provide a complete Data Availability Statement in the submission form, ensuring you include all necessary access information or a reason for why you are unable to make your data freely accessible. If your research concerns only data provided within your submission, please write "All data are in the manuscript and/or supporting information files" as your Data Availability Statement.**

**Response:** We have revised the Data Availability Statement in the submission form.

**5. When completing the data availability statement of the submission form, you indicated that you will make your data available on acceptance. We strongly recommend all authors decide on a data sharing plan before acceptance, as the process can be lengthy and hold up publication timelines. Please note that, though access restrictions are acceptable now, your entire data will need to be made freely accessible if your manuscript is accepted for publication. This policy applies to all data except where public deposition would breach compliance with the protocol approved by your research ethics board. If you are unable to adhere to our open data policy, please kindly revise your statement to explain your reasoning and we will seek the editor's input on an exemption. Please be assured that, once you have provided your new statement, the assessment of your exemption will not hold up the peer review process.**

**Response:** Following the journal’s recommendation, we have finalized our data sharing plan prior to acceptance. The revised Data Availability Statement reflects that all data required to reproduce the findings are already freely accessible within the manuscript and supporting materials. Therefore, no additional restrictions or exemptions are requested.

**6. Please amend either the title on the online submission form (via Edit Submission) or the title in the manuscript so that they are identical.**

**Response:** We have carefully checked the title and amended either the online submission form or the manuscript so that the titles are now fully identical.

**7. If the reviewer comments include a recommendation to cite specific previously published works, please review and evaluate these publications to determine whether they are relevant and should be cited. There is no requirement to cite these works unless the editor has indicated otherwise.**

**Response:** We have carefully reviewed all reviewer comments. The reviewers did not recommend citing any specific previously published works. Therefore, no additional references were added in response to this item.

**8. Please review your reference list to ensure that it is complete and correct. If you have cited papers that have been retracted, please include the rationale for doing so in the manuscript text, or remove these references and replace them with relevant current references. Any changes to the reference list should be mentioned in the rebuttal letter that accompanies your revised manuscript. If you need to cite a retracted article, indicate the article’s retracted status in the References list and also include a citation and full reference for the retraction notice.**

**Response:** We have thoroughly reviewed the entire reference list to ensure accuracy, completeness, and currency. No retracted articles are cited in the revised manuscript. Any necessary updates or corrections to references have been implemented and are noted in the rebuttal letter accompanying the revised submission.

**Replies to the reviewer**

**Reviewer 1**

The primary theoretical contribution of this study lies in proposing and validating a dual-mediation pathway model demonstrating how subjective movement experiences influence depressive symptoms in adolescents. The findings hold significant practical implications for the prevention and intervention of adolescent depression.

1. **The study has significant limitations: its cross-sectional design prevents establishing causal relationships or temporal sequences between variables. While the research identified an association between subjective exercise experiences and depressive symptoms, it cannot determine whether subjective exercise experiences influence depressive symptoms, depressive symptoms affect subjective exercise experiences, or if a bidirectional relationship exists.**

**Response:** Thank you for your important suggestions. We fully agree with your opinion and have supplemented and revised the content of the manuscript according to your suggestions.

Firstly, since the present study adopted a cross-sectional design, it can only identify the associations among subjective exercise experience, sleep quality, fatigue, and depressive symptoms based on the observed data. Although the proposed chain mediation model revealed significant relationships among these variables, causal inference and temporal ordering cannot be definitively established.

Secondly, through correlation analysis and structural equation modeling, significant direct and indirect paths were observed between subjective exercise experience and depressive symptoms via sleep quality and fatigue. The results showed that adolescents with more positive subjective exercise experiences tended to report better sleep quality, lower levels of fatigue, and fewer depressive symptoms. These findings indicate a consistent and theoretically meaningful pattern of associations, suggesting that subjective exercise experience is closely related to adolescents’ emotional health.

Finally, the identified chain mediation pathway—subjective exercise experience → sleep quality→fatigue→depressive symptoms—aligns well with existing theoretical frameworks and empirical evidence in the literature. From the perspective of the research theme, these findings suggest that interventions emphasizing positive exercise experiences, particularly those that contribute to improvements in sleep quality and reductions in fatigue, may have the potential to alleviate depressive symptoms among adolescents. Nevertheless, we acknowledge that longitudinal or experimental studies are required in future research to further verify the causal direction and stability of these relationships.

We have added a dedicated section on Limitations and Future Directions at the end of the conclusion. In this section, we explicitly note that the cross-sectional design prevents us from establishing causal relationships and acknowledge the potential reverse effects of depressive symptoms on subjective exercise experience and sleep quality. We also suggest that future studies employ longitudinal designs or cross-lagged panel models to address this issue and validate the causal direction of these relationships.

**2. The sample comprised 538 Chinese high school students, yet the study did not specify the sampling method or sampling frame, making it impossible to assess the representativeness of the sample. Additionally, cultural backgrounds, educational systems, and lifestyles among Chinese adolescents may differ from those in other countries, potentially limiting the generalizability of the findings.**

**Response:** Thank you for your suggestion. Based on your suggestion, we have made the following explanations.

Firstly, the original submission lacked sufficient details regarding the sampling procedure. We adopted a stratified cluster random sampling method. In the section "2.1 Participants and procedure", we have supplemented a detailed description of the sampling framework.

Thank you for this thoughtful comment. We fully agree that differences in cultural background, educational systems, and lifestyles among Chinese adolescents may limit the generalizability of the findings. We acknowledge this as an important consideration when interpreting the results, and we will explicitly address this issue in future studies by extending the proposed model to samples from different cultural and educational contexts.

**3. The use of self-administered questionnaires for assessment may introduce social desirability bias and recall bias. Particularly when assessing subjective exercise experiences, individual cognitive biases may compromise reporting accuracy.**

**Response:** Thank you for this valuable comment. We acknowledge that the use of self-administered questionnaires may introduce potential sources of bias, such as social desirability bias and recall bias, particularly when assessing subjective exercise experiences. Nevertheless, we would like to note that self-report questionnaires remain the most widely used and methodologically feasible approach for assessing psychological constructs such as subjective exercise experience, fatigue, sleep quality, and depressive symptoms. The instruments adopted in the present study have been extensively validated and applied in prior research, demonstrating acceptable reliability and construct validity across adolescent populations. Moreover, subjective exercise experience is inherently perceptual and experiential in nature, making self-report measures an appropriate and commonly accepted method for capturing individual evaluations.

At the same time, we recognize the limitations associated with this assessment approach. Accordingly, we have acknowledged this issue as a methodological limitation and suggest that future studies may benefit from incorporating multi-method assessment strategies, such as objective physical activity indicators, sleep monitoring, or informant-based reports, to further reduce potential reporting bias and enhance measurement accuracy.

**4.While the study proposes a dual-mediation pathway model, other crucial mediating variables—such as self-efficacy, social support, and coping strategies—may have been omitted. Furthermore, potential interactions and moderating effects among variables remain under-explored in this complex relationship.**

**Response:** We would like to express our sincere gratitude for your valuable comments and suggestions on our manuscript.

Firstly, this is a very insightful and valuable suggestion. We fully agree that the mechanisms underlying adolescent depression are highly complex, and that a single mediation model is unlikely to capture all relevant psychological, social, and contextual factors involved in this process.

Secondly, in the present study, we intentionally focused on a theoretically grounded and parsimonious chain mediation model involving subjective exercise experience, sleep quality, and fatigue, with the aim of clearly testing a core behavioral–physiological–emotional pathway. To ensure model stability, interpretability, and statistical robustness—particularly given the cross-sectional design—other potentially important variables such as self-efficacy, social support, and coping strategies were not included in the current analysis.

Thirdly, following the reviewer’s suggestion, we have explicitly acknowledged these potentially omitted variables in the Limitations and Future Directions section of the revised manuscript. We now propose that future studies extend the present model by incorporating self-efficacy, social support, and coping strategies as additional mediators or moderators, and by examining possible interaction effects among individual and environmental factors. Such extensions may help construct a more comprehensive and ecologically valid theoretical framework for understanding how subjective exercise experience influences depressive symptoms in adolescents.
